# Randomised controlled trial of Compensatory Cognitive Training and a Computerised Cognitive Remediation programme

**DOI:** 10.1186/s13063-020-04743-y

**Published:** 2020-09-29

**Authors:** Frances Dark, Ellie Newman, Victoria Gore-Jones, Veronica De Monte, Marta I. Garrido, Ilvana Dzafic

**Affiliations:** 1 Metro South Addiction and Mental Health Services, 228 Logan Road, Woolloongabba, Queensland Australia; 2 St Kilda Road Clinic, Community Adult Mental Health, Alfred Psychiatry, Melbourne, Australia; 3 Queensland Brain Institute, Centre for Advanced Imaging, Centre of Excellence for Integrative Brain Function, The University of Queensland, Brisbane, Queensland Australia; 4 Melbourne School of Psychological Sciences, The University of Melbourne, Melbourne, Australia

**Keywords:** Cognitive remediation, Schizophrenia, Cognitive functioning, Neurocognition

## Abstract

**Background:**

Compensation and adaptation therapies have been developed to improve community functioning via improving neurocognitive abilities in people with schizophrenia. Various modes of delivering compensation and adaptation therapies have been found to be effective. The aim of this trial is to compare two different cognitive interventions, Compensatory Cognitive Training (CCT) and Computerised Interactive Remediation of Cognition–Training for Schizophrenia (CIRCuiTS). The trial also aims to identify if mismatch negativity (MMN) can predict an individual’s response to the compensation and adaptation programmes.

**Methods:**

This study will use a randomised, controlled trial of two cognitive interventions to compare the impact of these programmes on measures of neurocognition and function. One hundred clinically stable patients aged between 18 and 65 years with a diagnosis of a schizophrenia spectrum disorder will be recruited. Participants will be randomised to either the CCT or the CIRCuiTS therapy groups. The outcome measures are neurocognition (BACS), subjective sense of cognitive impairment (SSTICS), social functioning (SFS), and MMN (measured by EEG) in people with schizophrenia spectrum disorders.

**Discussion:**

This trial will determine whether different approaches to addressing the cognitive deficits found in schizophrenia spectrum disorders are of comparable benefit using the outcome measures chosen. This has implications for services where cost and lack of computer technology limit the implementation and dissemination of interventions to address cognitive impairment in routine practice. The trial will contribute to the emerging evidence of MMN as a predictor of response to cognitive interventions.

**Trial registration:**

Australian New Zealand Clinical Trials Registry (ANZCTR) ACTRN12618000161224. Registered on 2 February 2018. Protocol version: 4.0, 18 June 2018.

## Introduction

### Background and rationale

Functional deficits (i.e. social skills, community functioning) are a core feature of schizophrenia. They represent key diagnostic criteria for the disorder that precede illness onset and are a strong predictor of outcome. These deficits do not respond to current psychopharmacology [1–3], thus underscoring the need to develop alternative interventions such as psychosocial treatments to address functional impairments.

Over the last 15 years, there has been renewed awareness of the impact of cognitive impairment in schizophrenia and the effect these deficits have on recovery and treatment outcomes [4, 5]. Compensation and adaptation therapies have been defined as “behavioural training-based intervention that aims to improve cognitive processes (attention, memory, executive functioning, social cognition or meta cognition with the goal of durability and generalisation)” [6]. A meta-analysis published in 2011 demonstrated that compensation and adaptation therapies had an overall effect size of 0.45 on global cognition with larger effect when combined with adjunctive rehabilitation (ES = 0.59) [6].

The research to date has not been able to refine who responds to cognitive compensation and adaptation therapies. There is interest in objective tests such as electroencephalography (EEG). EEG findings of attenuation in mismatch negativity (MMN) in auditory oddball tasks are one of the most robust and replicable neurophysiological markers of schizophrenia [7]. The MMN response occurs when a rare, unexpected sound is played in a repetitive sequence of sounds and is thought to reflect a brain response to an error in what is predicted. This sensory prediction error response has also been shown to relate to functional ability in people at risk for schizophrenia [8]. There are pilot studies indicating it may be able to predict and be an objective indicator of people who would benefit from psychosocial interventions [9–11]. Being able to have more precise information on who would respond to interventions, especially psychosocial interventions that require a weekly commitment over months, would benefit participants and not expose people to the potentially demoralising effect of attending a therapy that they were not able to benefit from [12].

Many cognitive remediation programmes are delivered via computer-based programmes often accessed online [6]. The cost of the infrastructure requirements of these programmes can pose a barrier to their implementation in some services. Pen and paper programmes to address cognitive deficits have been previously developed and evaluated [6]. This study is aimed at comparing a pen and paper cognitive adaption programme (CCT) [13–15] with a computer-based cognitive remediation programme, CIRCuiTS [16].

### Objectives

This study will use a randomised controlled trial to test the equivalence of CCT compared with CIRCuiTs to address the cognitive deficits of schizophrenia spectrum disorders. The primary objective is to examine the equivalence of CCT in improving neurocognitive deficits in people with schizophrenia spectrum disorders compared with CIRCuiTS. The secondary objective examines if CCT and/or CIRCuiTS are associated with objective and subjective improvement in community and cognitive functioning. In addition, we aim to determine whether MMN at baseline can be used to predict on an individual level, who might benefit from either treatment.

### Trial design

The design is a single-blind, randomised controlled trial to examine the equivalence of CCT and CIRCuiTS. The trial also aims to examine if MMN at baseline can predict response to the compensation and adaptation therapies and if MMN improves from baseline to post-treatment.

The study will include 100 clinically stable patients with a schizophrenia spectrum disorder. Participants will be randomised in a 1:1 ratio to join either the CCT group or the CIRCuiTS group. Participants will all be active cases in Metro South Addiction and Mental Health Services (community based). Participants in the study will continue to receive standard clinical care (i.e. there are no restrictions on medication or psychosocial interventions, apart from participants receiving therapies addressing neurocognition). These two interventions will be delivered by trained mental health staff once (CCT 2-h session) or twice (CIRCuiTS 1-h sessions) per week for 12 weeks. Groups will be based on a maximum of 6 participants per facilitator. Individual clinical assessments will be at baseline, at post-treatment, and at 3-month follow-up. Randomisation will be carried out using a computer-generated randomisation table.

## Method: participants, interventions, and outcomes

### Study setting

The study will be conducted in the Community Mental Health Centres within Metro South Addiction and Mental Health Services, Queensland, Australia.

### Eligibility criteria

The specified inclusion criteria are as follows: (1) aged between 18 and 65 years (inclusive), (2) fulfilling the clinical diagnosis of DSM-V criteria for schizophrenia spectrum disorder, (3) absence of uncorrected sensory impairments, (4) English literacy skills greater than grade 4 as per years of education, and (5) agreement to participate, with capacity to consent and able to follow the study instructions and procedures.

The specific exclusion criteria are as follows: (1) presence of substance dependence (with the exception of tobacco), (2) intellectual handicap (estimated using the Test of Premorbid Functioning (TOPF)), (3) people who are unable to understand or communicate in English or with English literacy skills less than grade 4 as per years of education, and (4) comorbid physical illnesses that would impair the participants’ ability to complete the trial.

### Interventions

All participants will continue in treatment as usual but will be asked not to be involved in other research or any therapy intervention aimed at improving their cognition until the study is completed. Participant withdrawal by the investigator will be determined by deterioration of mental state such that the participant is admitted to hospital or their ability to provide ongoing informed consent is compromised.

The investigator is responsible for the detection and documentation of events meeting the criteria and definition of an adverse event or serious adverse event as provided in this protocol. All adverse events will be recorded between the time of consent, intervention, and follow-up visits. Each participant will be monitored regularly by the investigator and study personnel for adverse events occurring throughout the study. The research team will enquire about adverse events by asking the following non-leading questions. At the first scheduled visit (baseline), participants will be asked:

“How are you feeling? Does your current treatment cause you regular side effects? Do you have any general health conditions that cause you problems on a regular basis (e.g. that we might expect to occur over the duration of this study)?”

At subsequent scheduled visits, participants will be asked:

“Since your last visit, have you had any health problems?”

Compensation and adaptation therapies will be delivered by two experienced CCT and CIRCuiTS therapists (FD and VDM) and co-facilitators. FD will facilitate the CCT groups and VDM will facilitate the CIRCuiTS groups. Supervision will be provided by the experienced therapists (FD and VDM). The therapy groups will be run at public mental health services community sites.

### Compensatory Cognitive Training

CCT is a 2-h ×12 session manualised cognitive compensatory training programme that focusses on the use of strategies to improve real-world cognitive functioning. The manual covers exercises in prospective memory, attention, memory, and executive functioning. There are some drill and practice of tasks, but the emphasis is on strategy use and incorporation into everyday activities. CCT is delivered through paper and pen tasks.

### CIRCuiTs

CIRCuiTS (Computerised Interactive Remediation of Cognition–Training for Schizophrenia) is a modular package including tasks of a wide range of cognitive functions (particularly executive function and memory), intended to allow therapy programmes to be flexibly designed to incorporate only relevant tasks. Prior to attempting a task, the participant needs to register how long they believe the task will take, how difficult the task is expected to be, and the strategy they intend to use. They receive computer feedback on these dimensions and re-rate how useful the strategies were with the goal of developing metacognitive knowledge and awareness. CIRCuiTS is run for 1 h twice a week and is delivered through a computer. The CIRCuiTS programme used consists of 40 stages. The tasks are progressively more difficult as the participant works through the programme. Twenty sessions are considered an adequate treatment exposure.

### Outcomes

A battery of validated clinical measures will be conducted at baseline, at post-treatment, and at 3-month follow-up. Raters who are blind to the randomisation of condition (registered psychologists) will complete the measures. The assessors instruct the participant not to reveal their group allocation.

The following measures will be used:

1. Brief Assessment of Cognition in Schizophrenia (BACS) [17] is an instrument that assesses the aspects of cognition found to be most impaired and most strongly correlated with outcome in patients with schizophrenia. This is the primary outcome measure.
2. Test of Premorbid Functioning (TOPF) [18] is a test of premorbid intelligence as estimated from reading ability.
3. Subjective Scale to Investigate Cognition in Schizophrenia (SSTICS) [19]. This measures the perception of cognitive abilities when completing everyday tasks.
4. Social Functioning Scale (SFS) [20] assesses areas of functioning that are crucial to the community maintenance of individuals with schizophrenia.
5. Brief Psychiatric Rating Scale (BPRS) [21] is a widely used scale for measuring symptom severity of patients with schizophrenia.
6. Electroencephalography (EEG). Participants will listen to a duration auditory oddball paradigm while their brain responses are measured with EEG. An auditory oddball paradigm consists of presentations of sequences of predictable sound stimuli (standard sounds) that are infrequently interrupted by unpredictable sound stimuli (deviant sounds). Sounds will be delivered via headphones and will vary in duration. All tones will be presented in a sound range that is comfortable for the participants. In the “volatility” auditory oddball task, the participants will be asked to pay attention to the sounds in order to judge the proportion of different sound types and rate their confidence on this judgement. This will allow us to assess the ability of participants to learn the statistical relationships of sound types, as well as the confidence on their own judgement. Prior to the experiment, the participants will be familiarised with the different sound types and trained with two short practice tasks. Participants will make responses using a computer keyboard and a mouse. The duration of the practice task will be 2 min, and the duration of the volatility auditory oddball task will be approximately 18 min, with a total duration of testing of approximately 20 min per participant, including breaks.

The TOPF, SSTICS, BPRS, and EEG are secondary outcome measures.

### Participant timeline

**Fig. 1:**
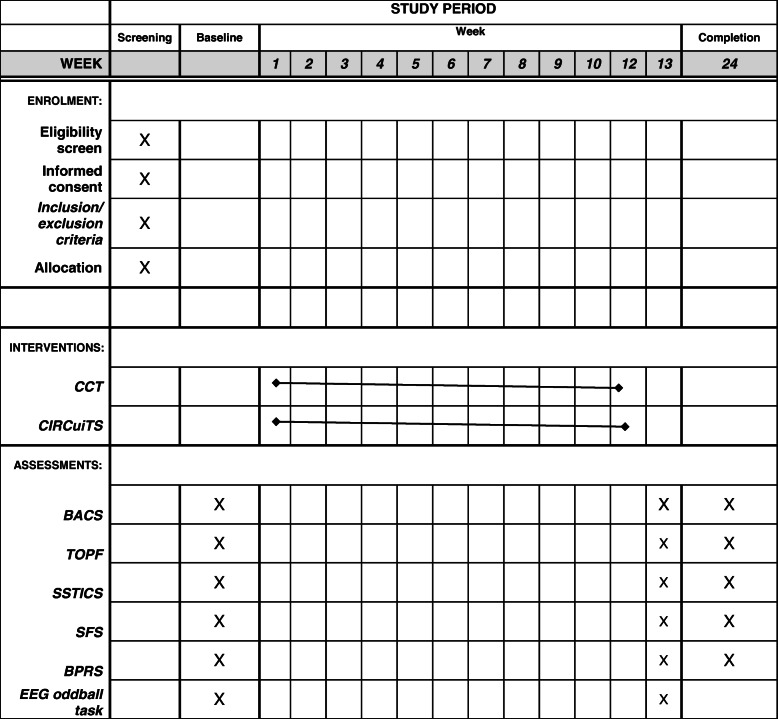
Schedule of enrolment, intervention, and assessment

The study will include clinically stable patients, living in the community with a diagnosis of a schizophrenia spectrum disorder receiving community-based clinical care in Metro South Addiction and Mental Health Services. These two interventions will be delivered by trained and experienced mental health staff once or twice per week for 12 weeks. Groups will be based on two to six participants per group. Individual clinical assessments will be at baseline, post-intervention, and 3-month follow-up (see Fig. 1).

### Sample size

In this equivalence study, we will focus on the primary outcome of cognition. We based our power analysis specifically on the BACS. We estimate a dropout rate of 15–20% (from treatment to 3-month follow-up) resulting in a final sample size of approximately 80 participants who will complete the study. This estimate is based on the dropout rates for studies with participants with serious mental illness as well as our experience with group randomised controlled trial research. Due to practicalities of group size and opportunities for running groups over the study timeframe, the maximum number of participants was set at one hundred.

### Recruitment

Participants will be recruited through the public mental health community clinics. Participants will continue to receive standard clinical care for the duration of the study. Medication prescribed at baseline will be collected and any changes noted. Participants will not be compensated for their time spent in treatment or assessment sessions.

## Methods: assignment of interventions

### Allocation and blinding

A minimum of twelve participants will be recruited for each round of the study. Participants will be recruited and randomly allocated in block sizes of four using randomisation procedure Statistical Analysis System (SAS) 9.4 by a member of the research team not involved in the delivery of the intervention or outcome assessment. As participants may drop out of therapy, the group numbers may fall to no lower than two participants. The therapeutic group size is two to six participants. Information about the treatment allocation will be emailed to the therapists who will contact participants about their therapy.

A battery of validated clinical measures will be conducted by trained research assistants, who are blind to group membership, at baseline, post-intervention, and 3-month follow-up. The research staff who undertake the outcome assessments will be blind to group allocation and will advise participants not to reveal their treatment. If a patient reveals this information, this will be noted for post hoc analyses of the data.

## Methods: data collection, management, and analysis

### Data collection and statistical methods

The primary efficacy analysis will assess average treatment group differences for the primary outcome measure BACS, over the entire study period (baseline, post-intervention, follow-up), and will use a likelihood based mixed-effects model, repeated measures (MMRM) approach. The MMRM model includes the fixed, categorical effects of treatment (CCT or CIRCuiTS), visit (baseline, 12 and 24 weeks), and treatment-by-visit interaction, as well as the continuous, fixed covariates of baseline score and baseline score-by-visit interaction. The MMRM includes all available data at each time point and is the preferred method of analysing clinical trial data in psychiatry as compared to more traditional repeated measures analysis of variance (ANOVA) and analysis of covariance (ANCOVA) models. Planned comparisons will be done with the MMRM models to determine between-group differences in change of symptoms measures from baseline to weeks 12 and 24. Results from the analysis of dichotomous data will be presented as proportions, with 95% confidence interval, and Fisher’s exact *p* value where appropriate. Non-parametric statistics will be used when assumptions for parametric methods are violated. Effect sizes will be calculated using Cohen’s guidelines. All tests of treatment effects will be conducted using a two-sided alpha level of 0.05 and 95% confidence intervals.

### EEG data collection and preprocessing

The EEG data will be collected with the AntNeuro EegoSport system, 64-electrode head cap at a sampling rate of 1024 Hz. The data will be downsampled to 200 Hz, high-pass filtered at 0.5 Hz, and low-pass filtered at 40 Hz using the Butterworth filter. The data will be epoched offline with a peri-stimulus window of − 100 to 400 ms, and baseline correction will be applied between − 100 and 0 ms. Next, artefact rejection will be performed by thresholding all channels at 100 uV and rejecting channels based on a 0.2-V threshold. We will then reference all the electrodes to the average reference. Finally, averaging will be conducted across all the trials. We will analyse event-related potentials from the onset of standard and oddball sounds, separately for stable and volatile conditions.

### Cognitive intervention-related changes in brain and behaviour: single-channel and behavioural analyses

To investigate if MMN improves after the compensation and adaptation therapy programmes, we will conduct a repeated measures 2 × 2 × 3 ANOVA design on mean ERP values, with MMN volatility (stable and volatile), treatment (CCT or CIRCuiTS), and visit (baseline, 12 and 24 weeks) as factors. Responders will be participants who make a clinically significant positive change based on the measures of cognition collected using Jacobson and Truax’s Reliable Change Index [22]. Mean ERP values will be obtained by averaging across the preselected time window of interest for MMN latency: 100–250 ms, over a frontocentral channel (Fz) [23]. In addition, we will investigate the behaviour, “regularity learning”, which is found to underlie the MMN response. We will conduct a separate repeated measures 2 × 2 × 3 ANOVA design with the following factors: regularity learning (stable and volatile), treatment (CCT or CIRCuiTS), and visit (baseline, 12 and 24 weeks). This behavioural analysis will be conducted to examine if the compensation and adaptation therapy programmes improve regularity learning ability. Significant interactions will be further analysed using paired *t* tests.

### Predicting cognitive intervention response with machine learning: spatiotemporal maps and feature definition

To identify if EEG measures can be used to predict response to the compensation and adaptation therapies, we will use machine learning techniques to classify individual patients that benefit from the compensation and adaptation therapies (responders) from patients who do not improve after the compensation and adaptation therapies (non-responders). Machine learning will be implemented using the Pattern Recognition for Neuroimaging Toolbox (PRoNTo) [23] with features from the whole-brain spatiotemporal analysis. First, we will create three-dimensional spatiotemporal images from averaged ERP data. A three-dimensional matrix (32 × 32 × 81) corresponding to the scalp electrode space and time points will be constructed, per participant for the different conditions. The images will be smoothed at full width half maximum of 12 mm × 12 mm × 20 ms. Before conducting the machine learning, we will perform the standard spatiotemporal mass-univariate general linear model analysis. A full factorial analysis will be performed to examine the spatiotemporal changes after the therapy programmes, with the following factors: volatility (stable and volatile), treatment (CCT or CIRCuiTS), and visit (baseline, 12 and 24 weeks). All statistical maps will be thresholded at *p* < 0.05 family-wise error (FWE) corrected for multiple comparisons.

Next, we will conduct the machine learning analysis, and the three-dimensional spatiotemporal images will be fed as features into the classifier. To classify the compensation and adaptation therapy responders and non-responders, class labels will be assigned to each participant. In PRoNTo, we will apply the support vector machine (SVM) and Gaussian process algorithms [24], which has shown to be effective in classifying schizophrenia patients using neuroimaging features [12]. The SVM training phase involves assigning weights to features and finding the hyperplane that maximises the margin between the groups of participants; the sign of the total feature weights multiplied by the test sample will determine the classification of participants. A *k*-fold cross-validation scheme will be conducted, dividing the group of participants into *k* subgroups, and iteratively assigning subgroups for training or testing until all subgroups have been through the testing. Mean-centering will be performed on all models and normalisation will be applied. In order to attain statistical measures, permutation tests will be performed (1000 repetitions) for each model and cross-validation scheme. To correct for multiple comparisons, we will apply false discovery rate correction of *q* = 0.05. Model sensitivity and specificity will be determined using the receiver operating characteristic curve, which will show the true positive rate (sensitivity) as a function of false positive rate (1 − specificity). The area under the curve will determine how well we are able to differentiate between compensation and adaptation therapy responders and non-responders using the best classifier from the EEG data.

Finally, we will conduct regression modelling to examine model performance by comparing true compensation and adaptation therapies’ response with that response predicted by the model. Spatiotemporal images for each subject will be mapped to their BACS scores, and machine learning regression algorithm—Kernel Ridge Regression [25, 26]—will be trained to predict these scores. To determine the accuracy of predicted response to compensation and adaptation therapies, we will calculate mean-squared error, Pearson’s correlation, and coefficient of determination statistics. Again *k*-fold cross-validation will be applied, permutations will be conducted to attain statistical measures, and multiple comparisons will be Bonferroni corrected.

### Data management

Researchers from Metro South Addiction and Mental Health Services will be responsible for data entry and analyses. Researchers from the Queensland Brain Institute will have access to the deidentified database to enter EEG data and perform analyses. Participants will be allocated a number to allow for their data from each time point to be linked for analyses.

A screening log will be utilised to track potential participants and record the number of individuals approached, consented, meeting inclusion/exclusion criteria, withdrawals, and completion (in keeping with standard CONSORT diagram requirements). Hard copies of the questionnaires, clinical assessments, and measures will be sent to the clinical co-ordinator and retained in a secure room, in a locked filling cabinet. The trial coordinator will be responsible for entering data into the password-protected database. The researchers will conduct the data analyses.

### Data monitoring

The trial management group (TMG) consists of the principal investigator (FD), the trial coordinator (VGJ), and associate investigator (VDM) who are unblinded to condition. Adverse events are reviewed by the TMG and reported to the ethics committee. The process of recruitment and data management are overseen by this group. This trial may be subject to random auditing by the ethics committee.

### Protocol amendments

Any amendments to the protocol will be submitted to the appointed HREC by the Chief Investigator for approval. Any approved amendments by the appointed HREC will be forwarded by the Chief Investigator for submission to the appropriate Research Governance Offices. If a protocol amendment requires changes to the informed consent form, the revised form will be approved by the reviewing ethics committees and site governance officers.

### Dissemination policy

Results will be disseminated in peer review publications and published international journals. Only deidentified data will be reported. Results will be provided to participants on their request.

## Discussion

Compensation and adaptation therapies have been demonstrated to improve neurocognitive functioning in people with schizophrenia spectrum disorders. The current study aims to evaluate the equivalence of two different compensation and adaptation therapies (CIRCuiTS and CCT) within a community mental health service. This study also aims to identify if mismatch negativity can predict an individual’s response to compensation and adaptation therapies. This knowledge will allow clinicians to identify individuals who are more likely to receive benefit from a compensation and adaptation therapy programme.

This RCT has several strengths. To date, no other study has incorporated neurophysiological measures of mismatch negativity to identify participants that may be more likely to benefit from compensation and adaptation therapies. This is a novel approach and may assist with identifying future participants of compensation and adaptation therapy programmes. Participants will be randomly assigned to each group, and the raters remain blind to condition to avoid bias. A variety of outcome measures will be used to assess functioning across neurocognitive, social, and neurophysiological domains.

No formal assessment of inter-rater reliability will be conducted. Groups will be matched for total exposure to therapy (2 h per week over 12 weeks). Participants in the CIRCuiTS group may not have completed all 40 phases prior to the post-intervention assessments. Clinician effects may influence the results as one therapist will deliver CCT and one will deliver CIRCuiTS.

## Supplementary information

**Additional file 1.**

## Data Availability

On completion of this study, the dataset will be made available from the corresponding author on reasonable request.

## Abbreviations

ANCOVA: Analysis of covariance
ANOVA: Analysis of variance
ANZCTR: Australian New Zealand Clinical Trials Registry
BACS: Brief Assessment of Cognition in Schizophrenia
BPRS: Brief Psychiatric Rating Scale
CIRCuiTS: Computerised Interactive Remediation of Cognition–Training for Schizophrenia
CCT: Compensatory Cognitive Training
CONSORT: Consolidated Standards of Reporting Trials
DSM-V: Diagnostic and Statistical Manual of Mental Disorders-Fifth Edition
EEG: Electroencephalography
FWE: Family-wise error
HREC: Human research ethics committee
IQ: Intelligence quotient
MMN: Mismatch negativity
MMRM: Mixed-effects model repeated measures
NDCT: National Database for Clinical Trials Related to Mental Illness
PRoNTo: Pattern Recognition for Neuroimaging Toolbox
RCT: Randomised controlled trial
SAS: Statistical Analysis System
SFS: Social Functioning Scale
SSTICS: Subjective Scale to Investigate Cognition in Schizophrenia
SVM: Support vector machine
TMG: Trial management group
TOPF: Test of Premorbid Functioning
