# Supplementary material for: Randomised controlled trial of Compensatory Cognitive Training and a Computerised Cognitive Remediation programme
Source: Trials. 2020 Sep 29;21:810. doi: 10.1186/s13063-020-04743-y (PMC7526389; doi:10.1186/s13063-020-04743-y)
Supplement: Supplementary file 1 — Additional file 1. [file 13063_2020_4743_MOESM1_ESM.doc]

**INFORMATION SHEET**

**Title** Randomized Controlled trial of are Cognitive Symptom Management versus Rehabilitation Therapy (Cogsmart) and Computerised Interactive Remediation of Cognition – Training for Schizophrenia (CIRCuiTS).

1. **Coordinating Principal Investigator** Dr Frances Dark

**Principal Investigator** Dr Frances Dark

**Location** Metro South Addiction and Mental Health Services

**Protocol COGSMART vs CIRCUITS**

**HREC Reference No:** AU/1/333D23

*This Participant Information and Consent Form is 8 pages long. Please make sure that you have all of the pages.*

**Introduction**

You are invited to take part in a multisite clinical trial of interventions aimed at improving thinking skills (e.g. memory and attention) which influence real world functioning. These skills can be affected by your illness. The aim of this clinical trial is to compare two therapies on their ability to improve thinking skills for those who have experienced psychosis.

Before you decide if you wish to consent to your participation we would like you to understand why the study is being done, what it will involve and how your information will be used. Please take time to read the following information carefully and if appropriate discuss it with friends, family and your doctor. One of our team will go through the information sheet with you and answer any questions you have. Please ask questions about anything that you do not understand or want to know more about.

Participation in this research is voluntary. If you don’t wish to take part, you don’t have to. It is desirable that your doctor be advised of your decision to participate in this study. If you have a doctor we strongly recommend that you inform them of your participation in this study. Once you understand what the project is about and if you agree to take part in it, you will be asked to sign the Consent Form. By signing the Consent Form, you indicate that you understand the information and that you give your consent to participate in the clinical trial. You will be given a copy of the Participant Information and Consent Form to keep as a record.

**What is the purpose of this study?**

Psychotic disorders include groups of illnesses that may affect a person’s thoughts, perceptions, emotions, social skills or communication. This study will look at which of the two cognitive therapies works best in improving thinking skills. The two therapies are Cognitive Symptom Management and Rehabilitation Therapy (Cogsmart) and Computerised Interactive Remediation of Cognition – Training for Schizophrenia (CIRCuiTS).

Cogsmart is a group based program that aims to improve real world thinking skills for functioning. It involves the use of strategies to help compensate for thinking difficulties. CIRCuiTS is a computerized program with modules including tasks of a wide range of cognitive functions (particularly problem solving and memory). It also includes”bridging” where participants in the group reflect on how the program relates to thinking skills needed in real world functioning. Both groups are equally as effective in helping with thinking skills.

Your participation in this trial will help us to determine if Cognitive Symptom Management and Rehabilitation Therapy or Interactive Remediation of Cognition – Training for Schizophreniaare worthwhile programs to offer as part of standard treatment to people who suffer from psychosis.

This research has been initiated by the Coordinating Principal Investigator, Dr Frances Dark who is employed at Metro South Hospital and Health Service.

**Do I have to take part in the study?**

No, you do not have to take part in this clinical trial. It is voluntary. If you decide to take part you will be given this Participant Information Sheet and asked to sign the attached Consent Form. You will be given a copy to keep for your records. If you decide to participate you can change your mind at any stage without this affecting your routine treatment or future health care.

**What does participation in the study involve?**

You are invited to take part in the clinical trial. You will be participating in a randomised controlled clinical trial. The term randomised indicates that you will be selected into either the Cognitive Symptom Management and Rehabilitation Therapy (Cogsmart) or Computerised Interactive Remediation of Cognition – Training for Schizophrenia (CIRCuiTS) group completely by chance (flip of a coin). This clinical trial will randomise participants in a 1:1 design, which means there is an equal chance of being in either the Cognitive Symptom Management and Rehabilitation Therapy (Cogsmart) or Computerised Interactive Remediation of Cognition – Training for Schizophrenia (CIRCuiTS) group.

**Step 1 Screening Process**

At first contact with a member of the research team, you will be asked to take part in an initial interview (screening interview) which will take approximately 30 minutes. If this screening process confirms that you can take part in the study, you will be consented and provided with the opportunity to enter the next phase of the study.

**Step 2 Contact**

Both programs involve participating in group sessions. You will be asked to attend group sessions either twice per week for 12 weeks, with each session lasting approximately 1 hour, or once per week for 12 weeks, with each lasting approximately 2 hours. Additionally, you will also be asked to attend three sessions as an individual – one at the beginning of the trial, one at the end of the trial and one at follow up 3 months later.

At each individual session a clinical trial research assistant/psychologist will conduct a series of clinical assessments which will take approximately 2 hours to complete. This will involve asking questions about your thinking and problem solving skills, thoughts, symptoms, and relationships.

It will also involve an EEG task. To do this task you will be seated in a comfortable chair and asked to listen to predictable and unpredictable (oddball) sounds, while wearing a cap with 64 electrodes, from which scalp electric brain activity will be recorded. You will listen to a sequence of predictable sounds that are infrequently interrupted by unpredictable sound stimuli. Sounds will be delivered via headphones and will vary in duration. All tones will be presented in a sound range that is comfortable for you. Prior to the task, you will be familiarised with the different sound types and trained with two short practice tasks. You make your responses using a computer keyboard and a mouse. The duration of the practice task will be 2min, the duration of the oddball task will be approximately 18 minutes, with a total duration of testing of approximately 20 minutes per participant, including breaks. The overall EEG assessment will take less than 90 minutes.

The research assistant/psychologist conducting the clinical assessments will be unaware (blind) of which group you are in. By keeping the research assistant unaware of which group you are in, and then comparing the Cognitive Symptom Management and Rehabilitation Therapy (Cogsmart) versus Computerised Interactive Remediation of Cognition – Training for Schizophrenia (CIRCuiTS) group, we will be able to learn if Cogsmart or CIRCuiTS improves thinking skills that help in every day functioning for people who suffer from psychosis. This type of evidence can help all patients to receive better treatments in the future.

**Will participating in the study cost me anything?**

There are no additional costs associated with participating in this clinical trial. The EEG will be conducted at the clinic that you attend for the groups.

**What are the possible benefits of taking part?**

We cannot guarantee or promise that you will receive any benefits from this clinical trial; however your participation will help us better understand if Cognitive Symptom Management and Rehabilitation Therapy (Cogsmart) and Computerised Interactive Remediation of Cognition – Training for Schizophrenia (CIRCuiTS) are effective in improves thinking skills that help in every day functioning for people who suffer from psychosis.

**What are the possible risks and disadvantages of taking part?**

There are some possible adverse effects or risks related to participation in this clinical trial which include:

1. Very occasionally, talking about mental illness can be upsetting. If by chance any of the group sessions or individual sessions causes you discomfort, you will not be expected to continue unless you wish to do so. If you do not want to continue or feel distressed or uncomfortable, a member of the clinical trial team will provide support to ensure your well-being. If you indicate a potential for self-harm or other serious risk to self or others, a member of the clinical trial team will report these responses immediately to the treating clinician.
2. Clinical Assessments. The main inconvenience is the time spent completing these tasks. If you find the testing tiring, you can have as many breaks as required or complete the assessments over two sessions.
3. Group sessions. The main inconvenience is the time spent participating in the activities. If you find the activities tiring, let your therapist know.
4. The EEG cap does not cause discomfort but is a new experience for you. The sounds are presented at a comfortable level. You are given practice runs with the tasks to ensure you feel comfortable. Please let the staff member conducting the EEG know if you feel anxious or need further explanation of the test.

**Can I have other treatments during this study?**

You will be able to remain on all of your current treatments whilst participating in the trial. We ask that during the study you do not participant in any group sessions related to Cognitive Symptom Management and Rehabilitation Therapy (Cogsmart) and Computerised Interactive Remediation of Cognition – Training for Schizophrenia (CIRCuiTS). But, you are able to continue your treatment as usual (medications and psychosocial treatments).

**What do I do if I wish to withdraw from the study?**

Your participation in this clinical trial is voluntary. You may choose not to participate, or you may decide to withdraw your consent and discontinue your participation from this trial at any time without affecting current or future care. If you wish to withdraw from this study please advise the clinical trial team. As part of consenting to this clinical trial, you agree that the data you provide will be used for the clinical trial if you decide to withdraw.

**What happens when the study ends?**

Once the study is completed, the results will be grouped together and comparisons will be made between Cognitive Symptom Management and Rehabilitation Therapy (Cogsmart) and Computerised Interactive Remediation of Cognition – Training for Schizophrenia (CIRCuiTS). These results will be published in a scientific journal and presented at scientific and community forums. You will be provided a summary of these results via a handout, which will be posted to you at the conclusion of the study,

**What will happen to information about me?**

Any information obtained in connection with this clinical trial that can identify you will remain confidential and will only be used for the purpose of this clinical trial and it will only be disclosed with your permission, except as required by law.

The information collected is classified as re-identifiable. This means that details that identify you have been removed from the information (by replacing this information with a code), but that is possible to link the code back to you if necessary. The code will be stored separately from the data. The information collected from you in this clinical trial will be entered into a database, using the code rather than your personal identifiable details. However, the clinical trial team, regulatory authorities, and Metro South Human Research Ethics Committee (HREC) and site Governance, will be able to inspect and have access to confidential data that identifies you by name. The researchers from the Queensland Brain Institute will only have access to your de-identified data. Any analysis, interpretation and publication of the study results will not identify you.

The paper files from your interviews and from the group’s sessions will be stored in locked filing cabinets in a dedicated research office. Computer files will be kept on a password-protected computer at a designated site (which has high level security). Only approved clinical trial staff, Metro South Human Research Ethics Committee and site Governance may access your data. Records relating to the results of the trial will be kept for 7 years. After the 7 year period your paper records will be shredded and destroyed and computer files deleted.

**How can I access my information?**

In accordance with relevant Australian and/or Queensland privacy and other relevant laws, you have the right to request access to the information collected and stored by the research team about you. You also have the right to request that any information with which you disagree be corrected. Please contact the research team member named in the question section of this document if you would like access to your information.

**Who is organising the study?**

This clinical trial is being overseen by the Coordinating Principal Investigator Dr Frances Dark.

**Who has reviewed the study?**

All research in Australia involving humans is reviewed by an independent group of people, called a Human Research Ethics Committee (HREC). This clinical trial has been reviewed and given approval by Metro South Human Research Ethics Committee and site Governance.

**How do I get more information?**

You should ask for any information you want. If you would like more information about the study or if there is any matter about it that concerns you, either now or in the future, do not hesitate to ask one of the members of the clinical trial team or your doctor. Before deciding whether or not to take part you may wish to discuss the matter with a relative or friend or with your doctor. You should feel free to do this.

If you have any questions about the study at any time, feel free to contact the researchers

Dr Frances Dark 3317 1129

Victoria Gore-Jones 3317 1015

Email: [Frances.Dark@health.qld.gov.au](mailto:Frances.Dark@health.qld.gov.au)

[Victoria_Gore-Jones@health.qld.gov.au](mailto:Victoria_Gore-Jones@health.qld.gov.au)

Postal Address PO BOX 709 Stones Corner, QLD 4120

1. **Ethical Guidelines and Independent Contact**

This study has been approved by Metro South Human Research Ethics Committee and local site Governance, which is an appropriately constituted HREC under the National Health and Medical Research Council of Australia.

If you have any complaints about any aspect of the clinical trial, the way it is being conducted or any questions you can contact the HREC Coordinator, Metro South Human Research Ethics Committee on 3443 8047 (phone); or [EthicsResearch.PAH@health.qld.gov.au](mailto:EthicsResearch.PAH@health.qld.gov.au) (email). All complaints will be treated in confidence, investigated fully and you will be informed of the outcome.

**Participant Consent Form**

**Study Title** Randomized Controlled trial of Cogsmart vs CIRCuiTS

- I have read (or had read to me), the Information Sheet and I understand the purpose of the clinical trial, what is involved, what data is being collected, any possible risks, inconveniences or discomforts involved, and what will be done with the data upon completion of the clinical trial.
- I have been given the time and opportunity to ask questions about the clinical trial and any

questions I have asked have been answered clearly and to my satisfaction. I have also been given the opportunity to discuss this clinical trial with a person not connected to the clinical trial.

- I understand that all information provided by me is treated as strictly confidential and will only be shared with the clinical trial team and not be released by the clinical trial team unless required to do so by law.
- I give permission for my doctors, other health professionals, hospitals or laboratories outside this hospital to release information to Metro South Hospital and Health Service concerning my disease and treatment for the purposes of this project. I understand that such information will remain confidential.
- I understand that research data gathered for the clinical trial will be published but will not be individually identifiable in any of these publications.
- I know that I may withdraw from the trial at any time without having to give any reason or affecting my current or future medical treatment.
- I understand I will receive a copy of the participant information and signed consent form to keep.
- I understand and consent to those regulatory authorities and other organisations referred to in the participant information having access to my confidential information.
- I agree to participate in this research and give my consent voluntarily.
- If I have completed the Test of Premorbid Functioning (TOPF) within the past year, I agree and consent the research team to access this data from other research studies or treating health professionals to form part of my baseline assessment.

**In addition (optional): (initial next to your response)**

- I give permission for a member of the clinical trial team to recontact me within 5 years of signing this consent, regarding possible participation in further mental health research. Yes No
- I give consent for the research team to review my health outcomes via my medical records (paper and electronic) and Health Research Databases during the course of the current study. Yes No

______________________________________________ ________________

Printed Name of Participant Initial

______________________________________________ ________________

Signature of Participant Date (participant to date)
